# Cognitive and Behavioral Functioning in Female Former Soccer Players: Results from the Head Impact and Trauma Surveillance Study (HITSS)

**DOI:** 10.3390/brainsci16090974

**Published:** 2026-09-15

**Authors:** Shania C. Mulayi, Anna Aaronson, Kelsey J. Goostrey, Fatima Tuz-Zahra, Yorghos Tripodis, William S. Cole-French, Matthew Roebuck, Greta Schneider, Brittany N. Pine, Joseph N. Palmisano, Brett M. Martin, Kenton H. Zavitz, Douglas I. Katz, Christopher J. Nowinski, Ann C. McKee, Thor D. Stein, R. Scott Mackin, Michael D. McClean, Jennifer Weuve, Jesse Mez, Michael W. Weiner, Rachel L. Nosheny, Michael L. Alosco, Robert A. Stern

**Affiliations:** 1 Boston University CTE Center, Boston University Chobanian & Avedisian School of Medicine, Boston, MA 02118, USAkjgoos11@bu.edu (K.J.G.); nowinski@concussionandcte.org (C.J.N.); tdstein@bu.edu (T.D.S.);; 2 Boston University Alzheimer’s Disease Research Center, Boston University Chobanian & Avedisian School of Medicine, Boston, MA 02118, USA; 3 Department of Biostatistics, Boston University School of Public Health, Boston, MA 02118, USA; 4 Department of Psychology, University of Wyoming, Laramie, WY 82071, USA; 5 Center for Health Data Science (CHDS), Boston University School of Public Health, Boston, MA 02118, USA; 6 Cambridge Cognition, Salt Lake City, UT 84101, USA; kenton.zavitz@camcog.com; 7 Department of Neurology, Boston University Chobanian & Avedisian School of Medicine, Boston, MA 02118, USA; 8 Department of Neurology, Boston Medical Center, Boston, MA 02118, USA; 9 Concussion and CTE Foundation, Boston, MA 02115, USA; 10 VA Bedford Healthcare System, US Department of Veterans Affairs, Bedford, MA 01730, USA; 11 VA Boston Healthcare System, US Department of Veterans Affairs, Jamaica Plain, Boston, MA 02130, USA; 12 Department of Pathology and Laboratory Medicine, Boston University Chobanian & Avedisian School of Medicine, Boston, MA 02118, USA; 13 Department of Psychiatry and Behavioral Sciences, University of California San Francisco, San Francisco, CA 94107, USA; 14 Department of Environmental Health, Boson University School of Public Health, Boston, MA 02118, USA; 15 Department of Epidemiology, Boston University School of Public Health, Boston, MA 02118, USA; jweuve@bu.edu; 16 Department of Radiology and Biomedical Imaging, University of California San Francisco, San Francisco, CA 94107, USA; 17 Department of Medicine, University of California San Francisco, San Francisco, CA 94107, USA; 18 Northern California Institute for Research and Education (NCIRE), San Francisco, CA 94121, USA; 19 Department of Anatomy & Neurobiology, Boston University Chobanian & Avedisian School of Medicine, Boston, MA 02118, USA; 20 Department of Neurosurgery, Boston University Chobanian & Avedisian School of Medicine, Boston, MA 02118, USA

**Keywords:** repetitive head impacts, soccer, females, cognition

## Abstract

**Highlights:**

**What are the main findings?**

- Among 2732 middle-aged women who played organized soccer earlier in life (including youth, high school, college, and semi-professional/professional levels), estimates of the cumulative amount of exposure to repetitive head impacts (e.g., from heading the ball) corresponded to a higher degree of current self-reported cognitive complaints, worse behavioral functioning, and more severe symptoms of depression.
- The number of self-reported lifetime concussions was associated with worse performance on only one computerized objective memory test.

**What are the implications of the main findings?**

- Continued longitudinal monitoring of the later-life brain health consequences of playing soccer among women is of critical importance.
- These findings may help to identify methods of minimizing the long-term effects of repetitive head impact exposure from playing soccer.

**Abstract:**

**Background/Objectives:** Repetitive head impacts (RHI) from contact and collision sports have been associated with later-life cognitive and neurobehavioral impairments, as well as neurodegenerative conditions such as chronic traumatic encephalopathy (CTE). RHI-associated clinical sequelae among female former soccer players, specifically, are not well understood. This cross-sectional study aimed to examine the relationship of RHI exposure proxies (e.g., total years of soccer play, concussion history, highest level of play, and estimated cumulative heading frequency) with clinical measures (e.g., subjective cognitive complaints, objective cognitive performance, behavioral dysregulation, and depressive symptoms) among 2732 women, aged 40 years or above, enrolled in the Head Impact and Trauma Surveillance Study (HITSS), all of whom formerly played organized soccer. **Methods:** HITSS participants completed an online battery that elicited self-reported cognitive and behavioral complaints and depressive symptoms and that assessed cognitive performance via computerized tests. Multivariable linear regression models estimated associations between soccer-related RHI proxies and outcome measures, adjusting for age and education. **Results:** Among the former soccer players, longer duration of soccer play, higher level of play, greater estimated cumulative heading frequency, and concussion history were significantly associated with worse self-reported cognitive functioning, greater behavioral dysregulation, and elevated depressive symptom severity. Apart from a single association between concussion history and visual learning and episodic memory performance, we found no other associations between RHI proxies and objective cognitive test performance. **Conclusions:** Among middle-aged women who played organized soccer, cumulative RHI exposure corresponded to a higher degree of subjective cognitive complaints, worse behavioral functioning, and more severe depressive symptoms. Continued monitoring of this large cohort of female former soccer players offers a more comprehensive understanding of long-term health consequences of soccer play.

## 1. Introduction

Exposure to repetitive head impacts (RHI) from contact and collision sports has been linked to later-life cognitive and behavioral impairments [1,2,3,4], as well as neurodegenerative diseases, including chronic traumatic encephalopathy (CTE) [5,6]. Most of this research has focused on men who have played American football. A growing body of work has been investigating neuropathological, cognitive, and neuropsychiatric sequelae of soccer play. CTE has been pathologically confirmed in former soccer players [7,8,9], and a 2023 study found that elite male soccer players had a significantly elevated risk of neurodegenerative disease compared to non-RHI-exposed controls [10]. In vivo studies, primarily of men, have also demonstrated associations between soccer play and cortical thinning, white matter disruptions [11,12], altered neurochemistry [13], and lower performance on neuropsychological tests, including measures of psychomotor speed and verbal memory [14].

Heading the soccer ball has been associated with cognitive deficits and neurophysiological changes among current and former soccer players, although findings have been mixed. Short-term exposure (e.g., immediately following a heading session, or heading in subsequent weeks or seasons) has been tied to poorer psychomotor speed performance and slower reaction times on executive function tasks [15], worse performance on a measure of working memory [16], and elevated blood-based biomarkers of neural damage (p-Tau217 and S100B) [17]. However, other studies found no acute deficits on an anticipation timing test [18] and no differences in concentrations of biomarkers of neuronal injury in individuals who headed the ball versus those who did not [19]. Long-term heading exposure has been linked to poorer verbal learning and memory [14], as well as white matter microstructural alterations [20], including lower fractional anisotropy in temporo-occipital white matter areas [21]. Additional studies have observed greater neurodegenerative disease risk among male former professional soccer players with longer career duration and those in non-goalkeeper positions [22], and former professional players also have higher neurodegenerative disease mortality compared to matched controls [23]. Quantifying risk in soccer play, and characterizing clinical symptoms associated with cumulative RHI exposure, however, remains challenging.

Most existing literature on the short and long-term consequences of soccer participation has focused on male soccer players, leaving female players comparatively under-included. Across all levels of play, female athletes have been shown to be at greater risk of sustaining head injuries [24,25]. In soccer specifically, studies have found that female players experience worse post-concussion outcomes, including more symptoms and poorer visual and verbal memory [26], as well as more extensive white matter microstructural alterations in association with heading the ball [27]. These differential responses may stem in part from sex-based anatomical differences, such as neck strength [28], as well as hormonal influences. For instance, fluctuations in estrogen and progesterone may modulate neuroinflammatory responses and cerebral metabolism post-head injury [29].

When comparing female soccer players to non-contact sport athletes, some studies have reported that soccer players perform significantly worse on measures of verbal memory, psychomotor speed, and verbal fluency [30,31]. However, other studies report no adverse cognitive effects when compared to controls [32]. For example, one study found that female soccer players exhibited normal age-related cognitive trajectories across multiple domains, including memory, psychomotor speed, reaction time, complex attention and cognitive flexibility and, in some cases, performed better than normative samples [33]. These mixed findings underscore the need for greater research focused specifically on cognitive and behavioral consequences of soccer participation among women.

Despite millions of female soccer players in the United States alone, no large-scale study has examined clinical correlates of estimated cumulative RHI exposure across all levels of play. To address this gap, the present study represents, to our knowledge, the largest investigation of middle-aged female former soccer players. We examined associations between self-reported proxies for RHI exposure (e.g., duration of soccer participation, highest level of soccer play, estimated cumulative heading frequency, and concussion history) and objective cognitive performance, subjective cognitive complaints, self-reported behavioral dysregulation, and depressive symptoms among a large cohort of women who played organized soccer, aged 40 years and older, whose highest level of play spanned youth, high school, collegiate, semi-professional, and professional play. We hypothesized that greater RHI exposure from soccer would be associated with poorer cognitive test performance, increased subjective cognitive decline, greater behavioral dysregulation, and elevated depressive symptoms.

## 2. Materials and Methods

### 2.1. Study Design and Sample

We drew data for this cross-sectional study from the Head Impact and Trauma Surveillance Study (HITSS). HITSS is a longitudinal, observational study examining cognitive and behavioral functioning in former soccer and American-style football players aged 40 years or older in the United States. HITSS is an extension of the University of California, San Francisco (UCSF) Brain Health Registry (BHR), an online Alzheimer’s disease and related dementias research registry for the recruitment and longitudinal monitoring of >105,000 adults [34,35]. This remote, longitudinal registry-based approach to assessing former contact and collision sport athletes has been applied in a companion HITSS publication of male former American football players using the same battery [36], as well as other studies in the field [37]. HITSS includes a remote online battery composed of questionnaires eliciting self-reported data on demographics, athletic and medical history, and cognitive and behavioral complaints; the battery also includes three computerized cognitive tests. The battery takes approximately 90 min to complete, and, every twelve months, HITSS invites participants to complete subsequent waves of assessments via automated email messages. Participants receive no monetary compensation for task completion but may enter a $500 gift card drawing. Recruitment of former soccer players was conducted via four channels: digital advertising (e.g., via Facebook and Instagram), organic media, such as press releases issued to large-scale media publications, in-person recruitment, and organizational partnerships. All participants provided written, electronic informed consent, and the study is approved by the Boston University Medical Campus Institutional Review Board. HITSS began enrolling participants in March 2022.

The present study consisted of data from a total of 2732 female former soccer players enrolled in HITSS. Their levels of play spanned a wide range: professional/semi-professional, college, high school, and youth. Participants were included if they self-identified as female, reported soccer as their primary sport (e.g., sport with the greatest duration of play), indicated that they no longer participated in soccer, and, at their baseline evaluation, responded to questions or completed tests related to at least one of the seven outcomes under investigation in this study. All included evaluation data were collected as of 9 April 2025. Participants were excluded if they played the following contact and collision sports: American football, hockey, boxing, futsal, rugby, wrestling, mixed martial arts, bull riding, equestrian, auto racing, horse jumping, or roller hockey. Completion rates by highest level of play were determined for each of the outcome measures. (Table 1).

### 2.2. Measures

#### 2.2.1. Measures of RHI Exposure

##### Boston University Repetitive Head Impact Exposure Assessment (RHIEA)

Investigators at Boston University developed the RHIEA to evaluate sports participation and other sources of RHI such as military service [38,39,40,41]. Participants are asked: “Have you ever participated in organized sports, which includes membership on a team with scheduled practices and games (excluding pick-up or neighborhood games)?” If the participant answers yes, they are asked additional questions regarding their participation in sports. If no, participants were directed to the next assessment; we excluded such participants from these analyses.

In this study, four metrics of RHI exposure were examined using the RHIEA: (1) Estimated cumulative heading frequency, derived by assigning numeric values to responses to the following question: “On average, compared to others who played the same position as you, how often did you head the ball?” Responses included: Less Frequently = 1, As Frequently = 2, and More Frequently = 3. These values were then summed across each participant’s total levels of play. This derivation included five levels of play (youth, high school, college, semi-professional and professional) and therefore ranged from 0 to 15. (2) Total years of play, which is defined as the total number of years the participant played organized soccer. (3) Highest level of play (i.e., youth [YTH], high school [HS], college [COL], or professional [PRO]), with professional defined as having played on a national team or in a professional or semi-professional league. (4) Concussion history, determined from participant responses to the following question: “As best as you can remember, how many total concussions did you have during your life?” To ensure accurate and standardized reporting, participants were provided with a clear and accessible definition of concussion: “a concussion has occurred anytime you have had a blow to the head that caused you to have symptoms for any amount of time. These include blurred or double vision, seeing stars, sensitivity to light or noise, headache, dizziness or balance problems, nausea, vomiting, trouble sleeping, fatigue, confusion, difficulty remembering, difficulty concentrating, or loss of consciousness. Whenever anyone gets a “ding” or their “bell rung,” that too is a concussion.” Responses were recorded as a continuous numeric variable of self-reported lifetime concussions.

#### 2.2.2. Subjective Cognitive Complaints

##### Behavior Rating Inventory of Executive Function—Adult Version (BRIEF-A) Metacognition Index (MI)

The BRIEF-A is a widely used measure of executive functioning and self-regulation in daily life, based on 75 self-report items [41]. These domains have been shown to be impaired in former American football players [1]. Participants rate each item on a 3-point Likert scale (1 = never, 2 = sometimes, 3 = often). There are nine clinical subscales, which are empirically validated and divided into two indices: Metacognition Index (MI) and Behavioral Regulation Index (BRI). The MI encompasses the Initiate, Working Memory, Plan/Organize, Organization of Materials, and Monitor clinical subscales. Age- and education-standardized T-scores for each clinical scale and the MI were used in this study. A total of 2312 participants (142 PRO, 740 COL, 1330 HS, 101YTH) completed this task.

##### Everyday Cognition Scale (ECog)

The ECog is a 39-question assessment designed to evaluate a participant’s perception of changes in their ability to perform daily tasks compared to ten years prior across six cognitive domains: memory, language, visuospatial abilities, planning, organization, and divided attention [42]. Online adaptations of the ECog have demonstrated validity for measuring cognitive decline and everyday functional abilities [43]. Participants rate each item on a 4-point Likert scale ranging from “better or no change” to “consistently much worse.” The ECog mean score for each participant was calculated by averaging their responses across all questions, with higher scores indicating greater self-reported cognitive decline. A total of 2725 participants (164 PRO, 884 COL, 1563 HS, and 114 YTH) completed this task.

#### 2.2.3. Objective Cognitive Performance (CANTAB)

##### Paired Associates Learning (PAL)

The PAL is part of the Cambridge Neuropsychological Test Automated Battery (CANTAB) and evaluates episodic memory and visual learning [44,45,46]. The task involves pairing different images and testing the participant’s ability to learn and recall the locations of various patterns. Over time, the difficulty increases as the number of patterns increases. The total number of errors and the first attempt memory score (FAMS), which measures the initial memory performance, are recorded to assess short-term memory and immediate recall [46]. An online version of the PAL was used, and 797 participants (46 PRO, 251 COL, 461 HS, and 39 YTH) completed this task.

##### One Touch Stockings (OTS)

The OTS [47] is another CANTAB test and is used to assess executive functioning, specifically spatial planning and problem-solving ability. During the test, participants view two arrangements of colored balls and must determine the minimum number of moves needed to match one to the other. Instead of manipulating the objects, they respond with a single touch, selecting how many moves would be required. The test measures planning ability, working memory, and decision-making under increasing levels of difficulty. This analysis examined OTS Problems Solved on First Choice (OTSPSFC), which is the total number of assessed trials in which the participant selected the correct answer on their first attempt. A total of 596 participants (24 PRO, 185 COL, 358 HS, 29 YTH) completed this task.

##### Spatial Working Memory (SWM)

The CANTAB SWM [47] test evaluates an individual’s ability to retain and manipulate spatial information, an aspect of executive function. Participants search for hidden tokens inside boxes on the screen while avoiding boxes already checked; this requires the use of spatial memory and strategy. As the task becomes more complex, it increasingly challenges the participant’s working memory capacity. For this study, we included two SWM scores: (1) SWM Strategy (SWMS), which is the number of times a participant began a new search pattern from the same box they previously started with. If they always begin a search from the same starting point, it can be inferred that the participant is using a pre-planned strategy to find tokens; (2) SWM Between Errors (SWMBE): the number of times the participant incorrectly revisited a box in which a token was previously found. This metric is calculated across all four, six and eight token trials. A total of 748 participants (39 PRO, 235 COL, 436 HS, 38 YTH) completed this task.

#### 2.2.4. Behavioral and Depressive Symptoms

##### BRIEF-A Behavioral Regulation Index (BRI)

The BRIEF-A Behavioral Regulation Index (BRI) [41] is derived from 30 self-report items assessing an individual’s ability to monitor their behavior and regulate impulses. This index includes the Inhibit, Shift, Emotional Control, and Self-monitor subscales. The scores for each clinical scale and index are summed, and for this study, the raw scores for each subscale and index were converted into T-scores. A total of 2312 participants (142 PRO, 740 COL, 1330 HS, 101YTH) completed this task.

##### Geriatric Depression Scale (GDS-15)

The GDS-15 is a self-report screening tool used to identify symptoms of depression in older adults [48]. This measure consists of 15 yes-or-no questions evaluating the emotional and cognitive aspects of depression. The number of “yes” responses is summed, with total scores ranging from 0 to 15. Normal scores range from 0 to 4, while scores ranging from 5 to 8 suggest mild depression, 9 to 11 indicate moderate depression, and 12 to 15 suggest severe depression. The GDS-15 has demonstrated good sensitivity and specificity among adults aged 18 and older [49]. A total of 2072 participants (127 PRO, 680 COL, 1174 HS, and 91 YTH) completed this task.

##### Mild Behavioral Impairment Checklist (MBI-C)

The MBI-C is a 34-item survey used to identify persistent behavioral and psychological change in older adults prior to cognitive decline and dementia [50]. The checklist assesses five key domains: (1) decreased motivation; (2) emotional dysregulation; (3) impulse dyscontrol; (4) social inappropriateness; and (5) abnormal perception or thought content. For each item, “yes” or “no” is used to indicate the presence of a symptom. If present, the symptom is rated based on severity using a 3-point Likert scale (1 = mild, 2 = moderate, 3 = severe). The total severity score is calculated by taking the sum of the severity for all endorsed symptoms. Higher scores indicate a greater risk for mild cognitive impairment, with a cut-off of 8.5 [51]. A total of 1916 participants (113PRO, 612 COL, 1113 HS, and 78 YTH) completed this task. The MBI-C is included in this study because individuals with RHI exposure and suspected CTE demonstrate neuropsychiatric features which overlap with MBI, often prior to age 50 [52,53,54]. The MBI-C was initially developed for individuals age ≥ 50. Although it has previously been used in studies with younger participants [55], its psychometric properties in younger samples have not yet been examined.

### 2.3. Statistical Analysis

All statistical analyses were performed using R version 4.5.1 and SPSS version 29.0.2.0. We fit multivariable-adjusted linear regression models to estimate mean differences in each outcome measure with increasing total years of soccer play and, in separate models, cumulative heading, and lastly, number of concussions. All estimates were adjusted for years of education. Analyses of outcomes expressed as raw scores were further adjusted for age in years, whereas analyses of outcomes expressed as age-standardized T-scores were not. Analyses of covariance (ANCOVA) models estimated associations between highest level of soccer play (YTH, HS, COL, PRO) and each outcome measure. For each ANCOVA model, an omnibus F-test assessed whether adjusted mean outcome scores differed jointly across the four levels of play. When the omnibus test was significant, post hoc Tukey-adjusted pairwise comparisons compared estimated marginal means of each outcome across each level of play.

*p*-values from the linear regression and ANCOVA models were adjusted by applying the Benjamini–Hochberg false discovery rate (FDR) method independently for each of the RHI proxies (level of play, years of play, estimated cumulative heading frequency, concussion history). The total number of hypotheses was set to two for the subjective cognitive domain (BRIEF-A MI, ECog), three for the mood/neurobehavioral domain (GDS, MBI-C, BRIEF-A BRI), and five for the objective cognitive (CANTAB) metrics (PAL FAMS, PALTEA, SWMS, SWMBE, OTSPSFC).

#### Sensitivity Analyses

All multivariable linear regression and ANCOVA models were performed excluding professional players (N = 164). Additional models were performed to examine associations of each RHI exposure metric with each BRIEF-A subscale (Inhibit, Shift, Emotional Control, Self-Monitor, Initiate, Working Memory, Plan/Organize, Task Monitor, and Organization of Materials). Multivariable linear regression models including all four RHI exposure metrics simultaneously were performed. Lastly, *p*-values for primary analyses were adjusted using a more conservative global Benjamini–Hochberg correction across all primary RHI–outcome tests rather than within domain × exposure families.

## 3. Results

### 3.1. Sample

**Figure 1:**
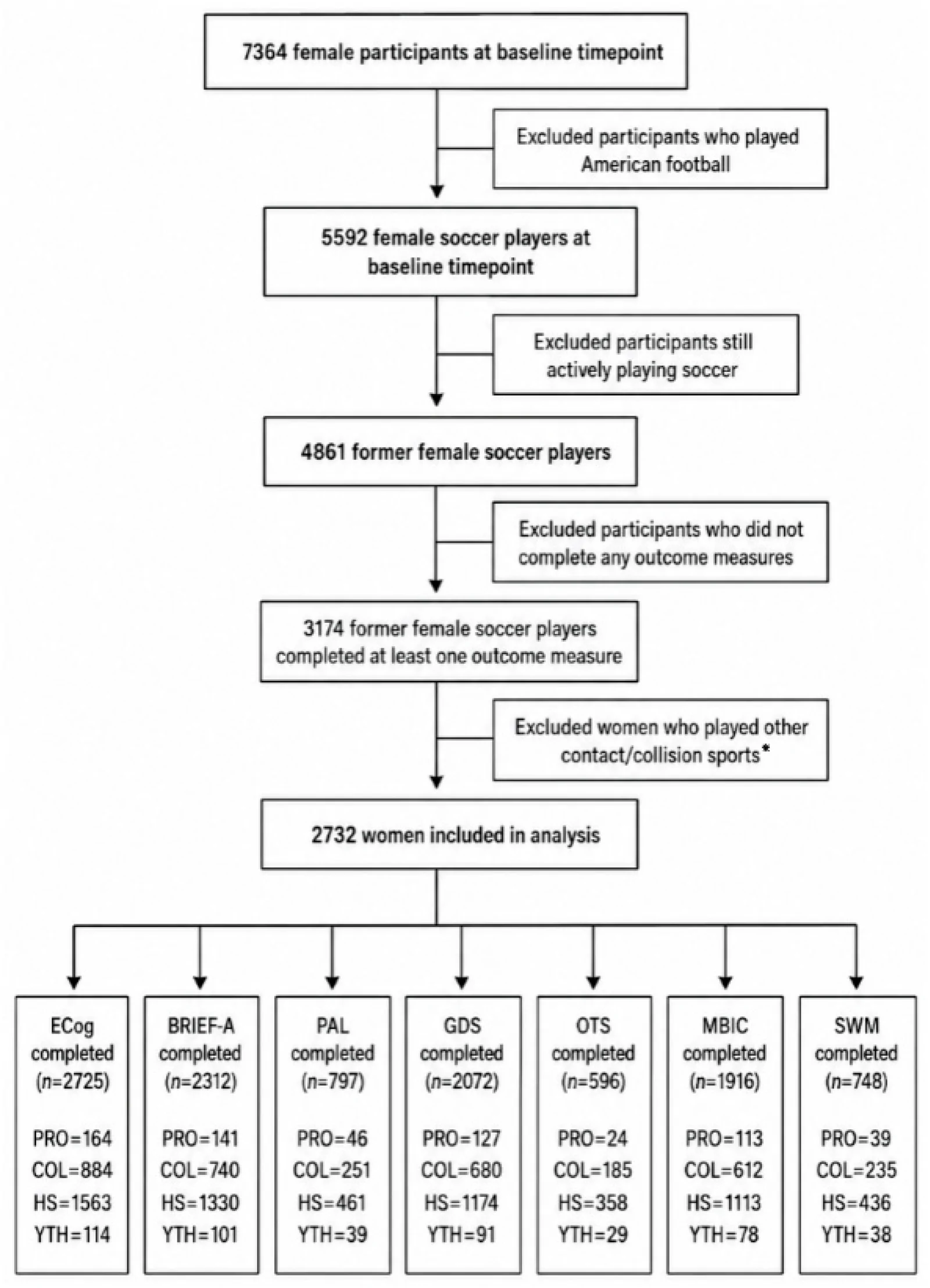
Sample derivation and task completion rates. Figure legend: Flow chart depicting derivation of the analytic sample, and individual task completion rates, stratified by highest level of soccer play. This figure includes rates of instrument/computerized test completion, meaning that the task was marked as “Collected” in the HITSS database. Numbers differ slightly from Table 1 and Table 2, which capture attempts at HITSS tasks (e.g., whether the task was started but not completed, or whether the participant encountered technical difficulties). Abbreviations: ECog = Everyday Cognition Scale, BRIEF-A = Behavior Rating Inventory of Executive Function—Adult, PAL = Paired Associates Learning, GDS = Geriatric Depression Scale-15, OTS = One Touch Stockings, MBI-C = Mild Behavioral Impairment Checklist, SWM = Spatial Working Memory, PRO = professional, COL = college, HS = high school, YTH = youth. * Other contact/collision sports include hockey, boxing, futsal, rugby, wrestling, mixed martial arts, bull riding, equestrian, auto racing, horse jumping, or roller hockey.

The soccer cohort (N = 2732) had a mean age of 47.08 years (SD = 5.91) and predominantly identified as white (2601/2715, 96%) and non-Latino (N = 2611/2704, 97%). Table 1 includes detailed sample characteristics. Table S1 includes participants’ race and ethnicity data. Sample derivation is described in Figure 1. There was only one significant association between the RHI proxies and performance on any of the CANTAB objective cognitive tests: concussion history and PAL FAMS. The following sections detail the findings on the self-report measures of subjective cognitive decline, depressive symptoms, and behavioral dysregulation.

**Table 2:** Associations between RHI exposures and cognitive/neurobehavioral outcomes.

Outcome	Predictor	*B* (95% CI) or *F*-Statistic	Adjusted *p*-Value ^1^
PAL FAMS	Highest level of play	*F*(3, 792) = 0.95	0.623
	Total years of play	0.00 (−0.05, 0.05)	0.988
	Cumulative heading	−0.01 (−0.10, 0.08)	0.908
	Concussion history	−0.07 (−0.11, −0.02)	0.015
PAL TEA	Highest level of play	*F*(3, 792) = 0.97	0.623
	Total years of play	0.02 (−0.10, 0.14)	0.988
	Cumulative heading	0.05 (−0.17, 0.28)	0.908
	Concussion history	0.12 (0.01, 0.23)	0.080
OTSPSFC	Highest level of play	*F*(3, 591) = 0.79	0.623
	Total years of play	−0.00 (−0.04, 0.03)	0.988
	Cumulative heading	0.04 (−0.03, 0.11)	0.661
	Concussion history	−0.01 (−0.05, 0.03)	0.739
SWM Between Errors	Highest level of play	*F*(3, 742) = 0.45	0.721
	Total years of play	0.15 (0.02, 0.28)	0.101
	Cumulative heading	0.01 (−0.22, 0.25)	0.908
	Concussion history	−0.03 (−0.14, 0.09)	0.739
SWM Strategy	Highest level of play	*F*(3, 742) = 2.21	0.430
	Total years of play	0.02 (0.00, 0.04)	0.101
	Cumulative heading	0.02 (−0.01, 0.06)	0.661
	Concussion history	−0.00 (−0.02, 0.01)	0.739
BRIEF-A MI	Highest level of play	*F*(3, 2324) = 1.08	0.355
	Total years of play	0.02 (−0.09, 0.12)	0.760
	Cumulative heading	0.39 (0.19, 0.60)	<0.001
	Concussion history	0.24 (0.18, 0.30)	<0.001
ECog Mean	Highest level of play	*F*(3, 2717) = 1.30	0.355
	Total years of play	0.00 (0.00, 0.01)	0.083
	Cumulative heading	0.02 (0.01, 0.03)	<0.001
	Concussion history	0.01 (0.01, 0.01)	<0.001
BRIEF-A BRI	Highest level of play	*F*(3, 2324) = 3.06	0.041
	Total years of play	0.14 (0.05, 0.23)	0.004
	Cumulative heading	0.57 (0.38, 0.75)	<0.001
	Concussion history	0.22 (0.16, 0.28)	<0.001
GDS-15	Highest level of play	*F*(3, 2075) = 3.79	0.030
	Total years of play	0.02 (0.01, 0.04)	0.002
	Cumulative heading	0.06 (0.03, 0.09)	<0.001
	Concussion history	0.02 (0.01, 0.03)	<0.001
MBI-C Total	Highest level of play	*F*(3, 1910) = 2.25	0.081
	Total years of play	0.17 (0.09, 0.25)	<0.001
	Cumulative heading	0.44 (0.28, 0.59)	<0.001
	Concussion history	0.18 (0.14, 0.23)	<0.001

^1^ *p*-values were false discovery rate corrected using the Benjamini–Hochberg method within domain × exposure. Abbreviations: GDS-15 = Geriatric Depression Scale-15, ECog = Everyday Cognition Scale, PAL FAMS = Paired Associates Learning First Attempt Memory Score, PAL TEA = Paired Associates Learning Total Errors Adjusted, BRIEF-A = Behavior Rating Inventory of Executive Function—Adult, MI = Metacognition Index, BRI = Behavioral Regulation Index.

### 3.2. Relationships Between RHI Proxies and Outcomes

#### 3.2.1. Level of Play

**Figure 2:**
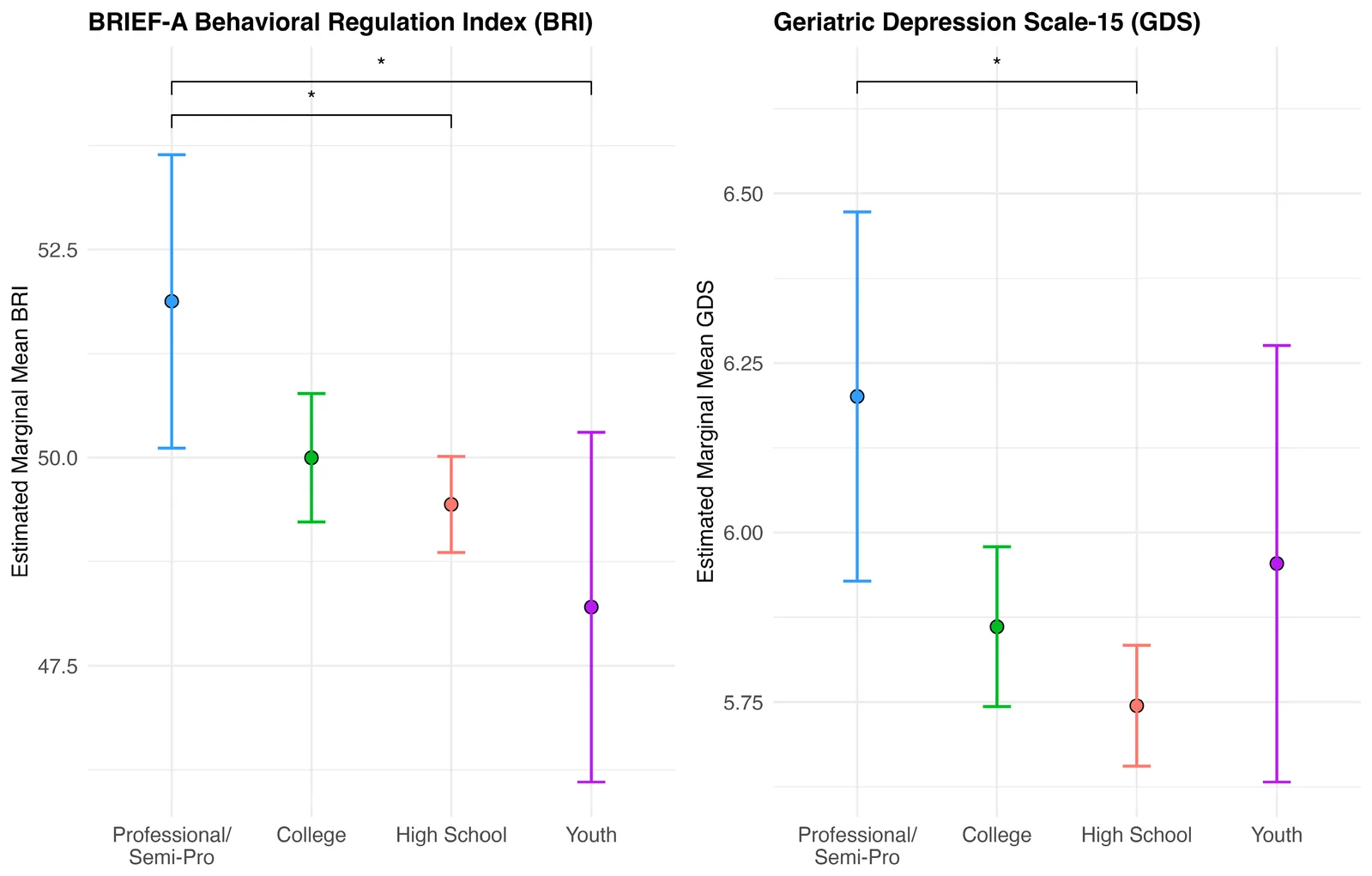
Mean BRI *T*-scores and GDS-15 (95% confidence interval) by highest level of soccer play. Figure Legend: Estimated marginal means of BRIEF-A Behavioral Regulation Index T-scores and Geriatric Depression Scale-15 (GDS-15) (95% confidence interval) by highest level of soccer play, adjusted for education. GDS-15 was additionally adjusted for age. An asterisk indicates a statistically significant difference (*p* < 0.05) from post hoc, pairwise Tukey-adjusted comparisons. Confidence intervals represent precision of group means across different levels of play. Abbreviations: BRIEF-A = Behavior Rating Inventory of Executive Function—Adult Version.

ANCOVA models demonstrated a significant omnibus effect for highest level of play on GDS-15 (*F* = 3.79, *p*_adj_ = 0.030) and BRIEF-A BRI (*F* = 3.06, *p*_adj_ = 0.041) (Table 2). Post hoc, Tukey-adjusted pairwise comparisons demonstrated that professional players scored significantly worse than high school players on both the GDS-15 (*p* = 0.010) and BRIEF-A BRI (*p* = 0.049) and also scored significantly worse than youth players on the BRIEF-A BRI (*p* = 0.043) (Figure 2).

#### 3.2.2. Years of Play

Multivariable linear regression models demonstrated significant associations between more total years of soccer play and higher (worse) scores across several neurobehavioral and cognitive outcomes. Specifically, longer duration of play was associated with greater behavioral dysregulation on the BRIEF-A BRI (*B* = 0.14, 95% CI = [0.05, 0.23], *p*_adj_ = 0.004). More years of play was also significantly associated with worse scores on the GDS-15 (*B* = 0.02, 95% CI = [0.01, 0.04], *p*_adj_ = 0.002) and the MBI-C (*B* = 0.17, 95% CI = [0.09, 0.25], *p*_adj_ < 0.001).

#### 3.2.3. Estimated Cumulative Heading Frequency

Greater estimated cumulative heading frequency was significantly associated with worse scores on the GDS-15 (*B* = 0.06, 95% CI = [0.03, 0.09], *p*_adj_ < 0.001), the MBI-C (*B* = 0.44, 95% CI = [0.28, 0.59], *p*_adj_ < 0.001) and the ECog (*B* = 0.02, 95% CI = [0.01, 0.03], *p*_adj_ < 0.001). Furthermore, estimated cumulative heading frequency was significantly associated with elevated scores on both global indices of the BRIEF MI (*B* = 0.39, 95% CI = [0.19, 0.60], *p*_adj_ < 0.001) and the BRIEF BRI (*B* = 0.57, 95% CI = [0.38, 0.75], *p*_adj_ < 0.001).

#### 3.2.4. Concussion History

A greater number of self-reported concussions was significantly associated with worse scores on the BRIEF-A MI (*B* = 0.24, 95% CI = [0.18, 0.30], *p*_adj_ < 0.001), the BRIEF-A BRI (*B* = 0.22, 95% CI = [0.16, 0.28], *p*_adj_ < 0.001), the ECog (*B* = 0.01, 95% CI = [0.01, 0.01], *p*_adj_ < 0.001), the GDS-15 (*B* = 0.02, 95% CI = [0.01, 0.03], *p*_adj_ < 0.001), and the MBI-C (*B* = 0.18, 95% CI = [0.14, 0.23], *p*_adj_ < 0.001). Concussion history was also significantly associated with worse performance on the PAL FAMS (*B* = −0.07, 95% CI = [−0.11, −0.02], *p*_adj_ = 0.015). No significant associations were observed between concussion history and PAL TEA, SWM Strategy, or SWM Between Errors (all *p*_adj_ > 0.05).

### 3.3. Results of Sensitivity Analyses

#### 3.3.1. Analyses Excluding Professionals

Excluding professional soccer players yielded smaller effect sizes for level of play, and associations with GDS-15 and BRIEF BRI lost significance altogether (Table 3). Associations with total years of play were diminished but remained significant for BRIEF-A BRI, GDS-15, and MBI-C, while effect sizes and significance patterns for estimated cumulative heading frequency were largely unchanged. The magnitude of the effect sizes for concussion history increased upon excluding the professional players, while retaining statistical significance; for example, *B* increased from 0.24 to 0.60 for the BRIEF-A MI and 0.18 to 0.51 for the MBI-C. Additionally, there was a significant association between concussion history and PAL TEA (*B* = 0.13, *p*_adj_ = 0.047). When *p*-values from primary models were adjusted via a more conservative global correction, all principal associations remained significant under this stricter correction, apart from the omnibus association between highest level of play and BRIEF-A BRI (*p*_adj_ = 0.064).

#### 3.3.2. Analyses of BRIEF Subscales

Exploratory multivariable linear regression models examining associations between the RHI exposure metrics and BRIEF scales demonstrated significant associations between concussion history and estimated cumulative heading frequency with each outcome measure. (Table 4). BRIEF Inhibit and Emotional Control were significantly associated with all four predictors.

#### 3.3.3. Models Including All RHI Metrics

Multivariable linear models incorporating all four RHI metrics simultaneously demonstrated significant associations of greater cumulative heading and concussion history with worse self-reported cognitive, neurobehavioral, and depressive outcomes. In contrast, neither highest level of play nor total years of play was independently associated with the inclusion of heading and concussion. Concussion history remained associated with computerized test performance (PAL FAMS, *B* = −0.07, *p* = 0.003; PAL TEA, *B* = 0.12, *p* = 0.033), whereas no exposure proxy was associated with the other computerized measures (Table S2).

## 4. Discussion

This study evaluated associations between proxies of RHI exposure in soccer and measures of cognition, subjective cognitive complaints, behavioral dysregulation and depressive symptoms among a large cohort of female former soccer players, aged 40 and older, spanning amateurs through professionals. We found small but statistically significant associations between RHI exposure and self-reported outcomes. Specifically, greater estimated cumulative heading frequency and greater concussion history were each associated with worse subjective cognitive complaints (ECog), self-reported behavioral dysregulation (BRIEF-A BRI and MI), and more severe depressive symptoms (GDS-15); higher level of play and more years of play were associated with a subset of these outcomes (e.g., BRIEF-A BRI and GDS-15). Representative effect sizes include cumulative heading and BRIEF-A BRI (*B* = 0.57, 95% CI 0.38–0.75) and concussion history and BRIEF-A MI (*B* = 0.24, 95% CI 0.18–0.30). No significant associations were found between RHI proxies and objective computerized cognitive test performance, apart from PAL FAMS and concussion history (*B* = −0.07, 95% CI −0.11 to −0.02). When professional players were excluded, significant associations between continuous RHI metrics and outcomes were maintained, though associations with highest level of play lost significance. To our knowledge, this represents the largest investigation of clinical symptoms and RHI exposure among middle-aged female former soccer players across all levels of play.

We found that both greater estimated cumulative heading frequency and increased concussions were associated with worse scores on most of the self-report measures of subjective cognitive complaints, depressive symptoms, and behavioral dysregulation, although overall effect sizes were small. Previous studies demonstrated adverse neurocognitive effects of heading the ball in soccer [56]. For example, Bruno et al. found that, among male former professional soccer players, higher estimated total career headers were associated with lower scores on Test Your Memory, a brief cognitive screening instrument [57]. Prior studies have also shown high prevalence of subjective cognitive complaints and decline among former athletes, including former football players [58] and female soccer players [31]. Furthermore, while numerous studies have observed associations between increased heading frequency and both acute and later-life impairments, the neurobiological mechanisms underpinning these impairments remain unclear. A recent study identified the orbitofrontal gray-white matter interface as a locus of RHI-related injury in soccer, associated with poorer verbal learning [59]. Similarly, a systematic review of sport-related concussion and neurocognitive outcomes among former athletes, including soccer players, revealed elevated self-report cognitive impairments [60]. Prospective, large-scale investigation into pathological changes associated with heading and concussion, and the association of these changes with clinical symptoms, is warranted.

We found some significant associations between higher level of soccer play as well as longer duration of play and elevations on self-report measures of behavioral dysregulation and depressive symptoms. Neurobehavioral dysregulation (e.g., explosivity, impulsivity, emotional lability) is a core clinical feature of traumatic encephalopathy syndrome (TES) [53], the proposed clinical syndrome associated with CTE pathology. Although depression is not a core clinical feature, it is one of several supportive features for determining levels of certainty for CTE pathology in the TES diagnostic framework. It is important to note, however, that TES features have not all been systematically verified against autopsy-confirmed CTE neuropathology. In a clinicopathological validation study of 336 donors, cognitive symptoms were significantly associated with CTE pathology, while mood and behavioral symptoms were not [61]. Neurobehavioral dysregulation, in isolation, therefore, does not reliably predict underlying CTE neuropathologic change. Neurobehavioral dysregulation has been examined in male football players [54,62,63,64,65] but has not yet been characterized in women at risk of CTE. This is crucial not only in terms of sex-based differential vulnerability to head impacts [66,67] but also in terms of discrepancies in how women experience and self-report symptoms. For example, Schaffert and colleagues found that emotional symptoms were a stronger predictor of self-reported cognitive symptoms than objective impairment among older retired female collegiate athletes [68]. In this vein, the relationship between depressive symptoms and subjective complaints warrants further consideration. Depression in middle age could be a major factor accounting for cognitive and behavioral complaints among this cohort. This underscores the need for longitudinal monitoring of disease trajectories.

We found few associations between RHI proxies and the computerized CANTAB tests used in this study as measures of objective cognitive impairment. This could be due to multiple factors, namely, that our cohort was young, mostly cognitively unimpaired, and exhibited no real objective impairments. Symptoms of any possible neurodegenerative conditions may not manifest until later in life. These findings diverge from our prior study of male former American football players enrolled in HITSS, which demonstrated associations of RHI metrics with poorer performance on CANTAB PAL, as well as increased depressive symptoms and subjective cognitive complaints. Additionally, within our cohort, there were relatively low completion rates for the CANTAB measures; therefore, there were insufficient data to rule out cognitive impairment. A recent study examining feasibility, acceptability, and construct validity of CANTAB PAL in BHR found that individuals with subjective memory concerns were less likely to complete the assessment [37]. Disproportionate completion of the measures could have biased our CANTAB findings toward the null.

Null findings and small effect sizes in our study could also stem from the ways in which RHI exposure was operationalized in the study. Unlike in American football, where helmet accelerometer data have validated metrics like the Cumulative Head Impact Index [2,69], soccer lacks such measures. Though there has been some usage of headbands and other sensors in soccer [70], there are no existing datasets that could provide adequate data by season for specific positions and levels of play. Additionally, total years of play is a crude metric for ascertaining exposure patterns in soccer, since there is substantial variability in terms of competitive structures and individual players’ total seasons played per year. RHI exposure in soccer is also heterogeneous. For example, heading frequency varies substantially across position of play, play style, and physical attributes (e.g., height), among other factors [71,72]. Consequently, the symptom burden observed in our cohort may be driven by a subset of athletes with high cumulative exposure, rather than signifying uniform risk. This heterogeneity could help explain why estimated cumulative heading frequency was a sensitive predictor in our models, as it may capture individual behavior more effectively than other RHI proxies. This is further illustrated by sensitivity analyses including all four RHI metrics simultaneously, in which concussion and cumulative heading retained independent associations with numerous measures, whereas level of play and years of play did not. Ultimately, this demonstrates that RHI exposure metrics used in football may not effectively quantify the spectrum of exposures in soccer.

## 5. Limitations

Our study has several limitations. Multiple selection biases limit the generalizability of our findings, including lack of ethnocultural and educational diversity in the sample, requirement for an internet-connected device and digital literacy, and underrepresentation of players whose highest level of play was youth. Recreational soccer play was excluded from the analysis, therefore eliminating an additional dimension of exposure. Only varsity college soccer players were included, not club or intramural players. Participants who played select contact and collision sports other than soccer were excluded; these sports could confer additional risks that were not examined in the present study. Importantly, this study failed to include an appropriate, non-RHI-exposed control group for comparison across outcomes. High prevalence of depressive symptoms and subjective cognitive concerns within this cohort could reflect selection bias for online Alzheimer’s disease and related dementias research registries. The HITSS battery was shortened part way through the study to decrease participant burden and improve retention. This impacted data collection. Finally, these findings are specific to soccer, in which heading is a sport-specific form of RHI; therefore, results may not be generalizable to other sports with different mechanisms or magnitudes of RHI exposure.

Only three CANTAB measures were administered in the HITSS battery, which assessed episodic memory and visual learning, spatial planning, and spatial working memory. There are limitations to unsupervised, computerized cognitive testing, including lack of free response and lack of supervision. We did not account for unmeasured, confounding factors such as family history of neurodegenerative diseases, physical and psychiatric comorbidities, and sociocultural and structural determinants of health, all of which could influence cognitive and neurobehavioral outcomes. Psychometric properties of the MBI-C have not yet been validated among cohorts younger than age 50, which warrants caution in interpretation of these findings. HITSS and BHR questionnaires were self-report and therefore may be subject to error and recall bias. Estimated cumulative heading frequency was operationalized as an ordinal measure, and future research is needed to determine whether heading frequency varies significantly across different levels of soccer play. Additionally, heading frequency was only assessed relative to other players in the same position. Future studies should also include estimates of the cumulative number of headers (e.g., per week, season). Lastly, missing data, especially relatively low completion rates for the CANTAB measures, could have limited power to detect significant effects. Lifetime concussion history was available for only 1597 of 2732 participants. Participants who provided concussion data may differ systematically from those who did not; therefore, findings may be subject to selection bias and may lack generalizability to the larger cohort.

It is important to consider the evolution of soccer conduct over the past several decades, including implementation of mandates to prohibit heading for players aged 10 and younger in the United States. Age of first exposure (AFE) has been examined as an RHI exposure metric primarily in American football. Few studies have examined AFE in soccer. One prior study included a small number of younger, active players and found no associations [73]. Interestingly, another study of adult amateur soccer players found that earlier AFE (≤10 years) was associated with better performance on working memory and verbal learning tasks in young adulthood, with no adverse associations observed [74]. However, AFE may be a flawed metric in soccer since first exposure to the sport may not coincide with AFE to RHI. Subsequent studies should collect information specifically about AFE to heading.

Another important consideration is the transition from leather soccer balls, which were prone to weight increase from water absorption [75] to hydrophobic synthetic balls beginning in the 1960s, but only becoming the dominant standard at elite levels in the 1990s. Furthermore, Title IX of the Education Amendments of 1972 (Title IX) mandated equal opportunities for men and women in sports. Since then, the percentage of female athletes has significantly increased [76,77] at the high school, college, and professional levels. Based on NCAA participation data, we estimate that more than 200,000 women have played college soccer since the passage of Title IX, with approximately 30,000 women currently participating annually across Divisions I, II, and III [78]. As the first wave of Title IX athletes age, the number of women at risk for aging-related neurologic conditions is expected to increase. Because of the slow growth of sports opportunities after the implementation of Title IX, studies of male and female soccer players may not be comparable due to exposure differences not captured by measures like years of play. The older women in this study lacked the same professional opportunities provided to men, both in length and intensity of their RHI exposure. In addition, the younger subjects in this study were more likely to play when soccer participation was year-round rather than only for one or two seasons a year, which may mask the cumulative effects of RHI and aging seen in other HITSS publications. Therefore, understanding later-life consequences of soccer play among women is of critical importance.

## 6. Conclusions

In conclusion, we found that, within a cohort of female former soccer players, there were significant associations between higher RHI exposure and self-reported cognitive, neurobehavioral, and depression-related outcomes. There was only one association between an RHI exposure metric and objective computerized neuropsychological test performance. Future analyses should examine more granular RHI exposure metrics, as well as longitudinal change in the neurocognitive and behavioral outcome measures, and whether these changes are associated with fluid and neuroimaging biomarkers of underlying pathophysiological change and neurodegenerative disease.

## Figures and Tables

**Table 1:** Soccer player characteristics by highest level of play.

			Levels of Play			
Characteristic	N	Overall *N* = 2732 ^1^	Youth *N* = 114 ^1^	High School *N* = 1567 ^1^	College *N* = 887 ^1^	Professional/Semi-Professional *N* = 164 ^1^
Age at Baseline	2732	47.08 (5.91)	47.89 (5.40)	47.05 (6.03)	47.00 (5.55)	47.29 (6.92)
Education	2731	17.32 (1.83)	17.25 (2.06)	17.28 (1.93)	17.39 (1.62)	17.31 (1.73)
Total years of soccer play	2732	9.11 (4.83)	3.77 (2.49)	7.63 (3.95)	11.16 (4.08)	15.82 (6.13)
Est. cumulative heading	2669	5.02 (2.40)	1.70 (0.78)	4.19 (1.78)	6.16 (1.98)	8.76 (3.03)
Lifetime concussions	1597	3.74 (9.87)	2.23 (5.09)	3.11 (5.62)	4.16 (6.99)	9.30 (34.01)
GDS-15 total	2081	5.82 (1.59)	5.98 (1.37)	5.74 (1.61)	5.86 (1.58)	6.20 (1.53)
ECog	2724	1.46 (0.44)	1.41 (0.40)	1.46 (0.44)	1.45 (0.44)	1.51 (0.47)
PAL FAMS	798	14.72 (3.34)	14.92 (3.31)	14.82 (3.28)	14.48 (3.45)	14.96 (3.40)
PAL TEA	798	8.51 (8.31)	7.90 (8.95)	8.23 (7.83)	9.15 (9.12)	8.28 (7.78)
BRIEF-A MI T-Score	2330	52.66 (12.40)	52.67 (12.63)	52.88 (12.37)	52.04 (12.23)	53.83 (13.33)
BRIEF-A BRI T-Score	2330	49.72 (10.83)	48.34 (10.07)	49.45 (10.54)	49.97 (11.13)	51.91 (12.05)
MBI-C total severity	1916	5.39 (8.13)	4.23 (6.91)	5.19 (7.87)	5.61 (8.38)	6.90 (9.69)

^1^ Mean (SD) for continuous variables; *n* (%) for categorical variables. Abbreviations: GDS-15 = Geriatric Depression Scale-15, ECog = Everyday Cognition Scale, PAL FAMS = Paired Associates Learning First Attempt Memory Score, PAL TEA = Paired Associates Learning Total Errors Adjusted, BRIEF-A = Behavior Rating Inventory of Executive Function—Adult, MI = Metacognition Index, BRI = Behavioral Regulation Index.

**Table 3:** Results of sensitivity analysis excluding professional players.

Outcome	Predictor	*B* (95% CI) or *F*-Statistic	Adjusted *p*-Value ^1^
PAL FAMS	Highest level of play	*F*(2, 747) = 1.08	0.532
	Total years of play	−0.00 (−0.06, 0.05)	0.941
	Cumulative heading	−0.03 (−0.13, 0.08)	0.622
	Concussion history	−0.07 (−0.12, −0.03)	0.006
PAL TEA	Highest level of play	*F*(2, 747) = 1.29	0.532
	Total years of play	0.02 (−0.12, 0.15)	0.941
	Cumulative heading	0.09 (−0.17, 0.35)	0.622
	Concussion history	0.13 (0.02, 0.25)	0.047
OTSPSFC	Highest level of play	*F*(2, 568) = 0.63	0.532
	Total years of play	−0.01 (−0.05, 0.03)	0.941
	Cumulative heading	0.03 (−0.05, 0.10)	0.622
	Concussion history	−0.01 (−0.04, 0.03)	0.746
SWM Between Errors	Highest level of play	*F*(2, 704) = 0.65	0.532
	Total years of play	0.17 (0.02, 0.31)	0.110
	Cumulative heading	0.07 (−0.20, 0.33)	0.622
	Concussion history	−0.03 (−0.14, 0.08)	0.746
SWM Strategy	Highest level of play	*F*(2, 704) = 3.31	0.185
	Total years of play	0.02 (−0.00, 0.04)	0.255
	Cumulative heading	0.02 (−0.02, 0.06)	0.622
	Concussion history	−0.00 (−0.02, 0.01)	0.746
BRIEF-A MI	Highest level of play	*F*(2, 2182) = 0.98	0.513
	Total years of play	−0.03 (−0.14, 0.09)	0.652
	Cumulative heading	0.39 (0.15, 0.63)	0.002
	Concussion history	0.60 (0.49, 0.72)	<0.001
ECog Mean	Highest level of play	*F*(2, 2554) = 0.67	0.513
	Total years of play	0.00 (−0.00, 0.01)	0.296
	Cumulative heading	0.02 (0.01, 0.03)	<0.001
	Concussion history	0.02 (0.02, 0.02)	<0.001
BRIEF-A BRI	Highest level of play	*F*(2, 2182) = 1.51	0.330
	Total years of play	0.11 (0.01, 0.21)	0.042
	Cumulative heading	0.56 (0.35, 0.77)	<0.001
	Concussion history	0.57 (0.47, 0.67)	<0.001
GDS-15	Highest level of play	*F*(2, 1949) = 1.68	0.330
	Total years of play	0.02 (0.00, 0.04)	0.039
	Cumulative heading	0.06 (0.03, 0.09)	<0.001
	Concussion history	0.05 (0.04, 0.07)	<0.001
MBI-C Total	Highest level of play	*F*(2, 1798) = 1.38	0.251
	Total years of play	0.15 (0.06, 0.23)	0.002
	Cumulative heading	0.44 (0.27, 0.61)	<0.001
	Concussion history	0.51 (0.44, 0.58)	<0.001

^1^ *p*-values were false discovery rate corrected using the Benjamini–Hochberg method within domain × exposure. Abbreviations: GDS-15 = Geriatric Depression Scale-15, ECog = Everyday Cognition Scale, PAL FAMS = Paired Associates Learning First Attempt Memory Score, PAL TEA = Paired Associates Learning Total Errors Adjusted, BRIEF-A = Behavior Rating Inventory of Executive Function—Adult, MI = Metacognition Index, BRI = Behavioral Regulation Index.

**Table 4:** Exploratory BRIEF subscale results.

Outcome	Predictor	*B* (95% CI) or F-Statistic	*p*-Value
BRIEF-A Organization of Materials	Highest level of play	*F*(3, 2324) = 0.52	0.671
	Total years of play	−0.01 (−0.11, 0.09)	0.786
	Cumulative heading	0.24 (0.04, 0.44)	0.021
	Concussion history	0.18 (0.12, 0.24)	<0.001
BRIEF-A Task Monitor	Highest level of play	*F*(3, 2324) = 2.19	0.088
	Total years of play	−0.01 (−0.11, 0.09)	0.822
	Cumulative heading	0.31 (0.12, 0.51)	0.002
	Concussion history	0.21 (0.15, 0.27)	<0.001
BRIEF-A Plan/Organize	Highest level of play	*F*(3, 2323) = 1.55	0.200
	Total years of play	0.00 (−0.09, 0.10)	0.922
	Cumulative heading	0.32 (0.12, 0.51)	0.001
	Concussion history	0.22 (0.16, 0.28)	<0.001
BRIEF-A Working Memory	Highest level of play	*F*(3, 2324) = 0.86	0.463
	Total years of play	0.08 (−0.03, 0.19)	0.146
	Cumulative heading	0.57 (0.36, 0.79)	<0.001
	Concussion history	0.24 (0.17, 0.30)	<0.001
BRIEF-A Initiate	Highest level of play	*F*(3, 2324) = 1.59	0.190
	Total years of play	0.02 (−0.08, 0.12)	0.732
	Cumulative heading	0.29 (0.09, 0.49)	0.005
	Concussion history	0.19 (0.13, 0.25)	<0.001
BRIEF-A Self-Monitor	Highest level of play	*F*(3, 2323) = 1.50	0.212
	Total years of play	0.06 (−0.02, 0.14)	0.133
	Cumulative heading	0.34 (0.18, 0.50)	<0.001
	Concussion history	0.18 (0.13, 0.22)	<0.001
BRIEF-A Shift	Highest level of play	*F*(3, 2324) = 1.34	0.260
	Total years of play	0.07 (−0.02, 0.16)	0.137
	Cumulative heading	0.44 (0.26, 0.63)	<0.001
	Concussion history	0.17 (0.12, 0.23)	<0.001
BRIEF-A Inhibit	Highest level of play	*F*(3, 2324) = 2.70	0.044
	Total years of play	0.14 (0.05, 0.22)	0.001
	Cumulative heading	0.51 (0.34, 0.68)	<0.001
	Concussion history	0.20 (0.15, 0.25)	<0.001
BRIEF-A Emotional Control	Highest level of play	*F*(3, 2324) = 3.07	0.027
	Total years of play	0.14 (0.05, 0.24)	0.002
	Cumulative heading	0.49 (0.31, 0.68)	<0.001
	Concussion history	0.17 (0.11, 0.23)	<0.001

BRIEF-A = Behavior Rating Inventory of Executive Function—Adult Version.

## Data Availability

De-identified data can be requested from Federal Interagency Traumatic Brain Injury Research (FITBIR) Informatics System: https://fitbir.nih.gov/content/access-data (accessed on 9 September 2026).

## Supplementary Materials

The following supporting information can be downloaded at: https://www.mdpi.com/article/10.3390/brainsci16090974/s1, Table S1: Participants’ Race and Ethnicity Data; Table S2: Mutually adjusted regression models including all RHI metrics.

## Abbreviations

AFE	Age of first exposure
ANCOVA	Analysis of covariance
BHR	Brain Health Registry
BRI	Behavioral Regulation Index (BRIEF-A)
BRIEF-A	Behavior Rating Inventory of Executive Function—Adult version
CANTAB	Cambridge Neuropsychological Test Automated Battery
CI	Confidence interval
COL	College (highest level of play)
CTE	Chronic traumatic encephalopathy
ECog	Everyday Cognition Scale
FAMS	First Attempt Memory Score
FDR	False discovery rate
FITBIR	Federal Interagency Traumatic Brain Injury Research
GDS-15	Geriatric Depression Scale-15
HITSS	Head Impact and Trauma Surveillance Study
HS	High school (highest level of play)
IRB	Institutional Review Board
MBI-C	Mild Behavioral Impairment Checklist
MI	Metacognition Index (BRIEF-A)
OTS	One Touch Stockings
OTSPSFC	One Touch Stockings Problems Solved on First Choice
PAL	Paired Associates Learning
PAL FAMS	Paired Associates Learning First Attempt Memory Score
PAL TEA	Paired Associates Learning Total Errors Adjusted
PRO	Professional/semi-professional (highest level of play)
HI	Repetitive head impacts
RHIEA	Boston University Repetitive Head Impact Exposure Assessment
SD	Standard deviation
SWM	Spatial Working Memory
SWMBE	Spatial Working Memory Between Errors
SWMS	Spatial Working Memory Strategy
TE	Traumatic encephalopathy syndrome
S	Title IX of the Education Amendments of 1972
Title IX	University of California, San Francisco
UCSF	Youth (highest level of play)
YTH	Age of first exposure
