# Supplementary material for: Cognitive and Behavioral Functioning in Female Former Soccer Players: Results from the Head Impact and Trauma Surveillance Study (HITSS)
Source: Brain Sci. 2026 Sep 15;16(9):974. doi: 10.3390/brainsci16090974 (PMC13604785; doi:10.3390/brainsci16090974)
Supplement: Supplementary file 1 [file brainsci-16-00974-s001.zip › brainsci-4485952-supplementary.pdf]

Supplemental Table S1: Participants' Race and Ethnicity Data

		Levels of Play				
**Characteristic**	**N**	**Overall** N = 2,732[1]	**Youth** N = 114[1]	**High School** N = 1,567[1]	**College** N = 887[1]	**Professional/Semi-Professional** N = 164[1]
**Race**	2,715					
African American/Black		6 (0.2%)	0 (0%)	2 (0.1%)	3 (0.3%)	1 (0.6%)
Asian		14 (0.5%)	1 (0.9%)	11 (0.7%)	1 (0.1%)	1 (0.6%)
Native American		4 (0.1%)	0 (0%)	4 (0.3%)	0 (0%)	0 (0%)
White		2,601 (96%)	112 (98%)	1,482 (95%)	854 (97%)	153 (94%)
Other		21 (0.8%)	1 (0.9%)	13 (0.8%)	4 (0.5%)	3 (1.9%)
Multiple		69 (2.5%)	0 (0%)	45 (2.9%)	20 (2.3%)	4 (2.5%)
**Ethnicity**	2,704					
Latino		93 (3.4%)	1 (0.9%)	60 (3.9%)	28 (3.2%)	4 (2.5%)
Not Latino		2,611 (97%)	113 (99%)	1,491 (96%)	850 (97%)	157 (98%)

[1]Cells display n (%)

Supplemental Table S2: Mutually adjusted regression models including all RHI metrics

Outcome	Predictor	B	95% CI	p
BRIEF-A BRI (T-score)	Level of play: High School (vs Youth)	1.75	(-1.19, 4.68)	0.243
	Level of play: College (vs Youth)	0.42	(-2.82, 3.65)	0.801
	Level of play: Professional/Semi-Pro (vs Youth)	0.65	(-3.58, 4.89)	0.763
	Years of play	-0.01	(-0.15, 0.14)	0.917
	Cumulative heading	0.56	(0.25, 0.87)	<0.001
	Concussion history	0.21	(0.15, 0.26)	<0.001
	Education	-0.38	(-0.69, -0.07)	0.015
BRIEF-A MI (T-score)	Level of play: High School (vs Youth)	1.28	(-2.08, 4.64)	0.453
	Level of play: College (vs Youth)	-0.93	(-4.63, 2.77)	0.622
	Level of play: Professional/Semi-Pro (vs Youth)	-0.52	(-5.37, 4.32)	0.832
	Years of play	-0.09	(-0.26, 0.07)	0.283
	Cumulative heading	0.55	(0.19, 0.91)	0.003
	Concussion history	0.23	(0.17, 0.30)	<0.001
	Education	-0.32	(-0.67, 0.04)	0.080
ECog mean	Level of play: High School (vs Youth)	0.03	(-0.08, 0.14)	0.621
	Level of play: College (vs Youth)	-0.02	(-0.15, 0.10)	0.717
	Level of play: Professional/Semi-Pro (vs Youth)	-0.02	(-0.18, 0.14)	0.787
	Years of play	-0.00	(-0.01, 0.00)	0.376
	Cumulative heading	0.02	(0.01, 0.03)	<0.001
	Concussion history	0.01	(0.01, 0.01)	<0.001
	Age	-0.00	(-0.01, 0.00)	0.275
	Education	-0.02	(-0.03, -0.01)	0.004
GDS-15 total	Level of play: High School (vs Youth)	-0.33	(-0.77, 0.12)	0.151
	Level of play: College (vs Youth)	-0.42	(-0.92, 0.07)	0.091
	Level of play: Professional/Semi-Pro (vs Youth)	-0.12	(-0.76, 0.53)	0.724
	Years of play	0.01	(-0.01, 0.03)	0.448
	Cumulative heading	0.04	(-0.00, 0.09)	0.068
	Concussion history	0.02	(0.01, 0.03)	<0.001
	Age	-0.01	(-0.02, 0.01)	0.246
	Education	-0.10	(-0.15, -0.05)	<0.001
MBI-C total	Level of play: High School (vs Youth)	0.27	(-2.09, 2.64)	0.821
	Level of play: College (vs Youth)	-0.61	(-3.21, 1.98)	0.643
	Level of play: Professional/Semi-Pro (vs Youth)	-1.34	(-4.72, 2.04)	0.437
	Years of play	0.07	(-0.05, 0.18)	0.273
	Cumulative heading	0.44	(0.19, 0.70)	<0.001
	Concussion history	0.17	(0.13, 0.22)	<0.001
	Age	-0.05	(-0.12, 0.03)	0.216
	Education	-0.41	(-0.66, -0.16)	0.001

Outcome	Predictor	B	95% CI	p
PAL FAMS	Level of play: High School (vs Youth)	-0.64	(-1.96, 0.68)	0.343
	Level of play: College (vs Youth)	-0.79	(-2.27, 0.69)	0.294
	Level of play: Professional/Semi-Pro (vs Youth)	0.01	(-2.04, 2.05)	0.994
	Years of play	-0.02	(-0.10, 0.06)	0.650
	Cumulative heading	0.07	(-0.08, 0.21)	0.387
	Concussion history	-0.07	(-0.11, -0.02)	0.003
	Age	-0.17	(-0.21, -0.12)	<0.001
	Education	0.02	(-0.15, 0.18)	0.854
PAL TEA	Level of play: High School (vs Youth)	2.39	(-0.96, 5.73)	0.162
	Level of play: College (vs Youth)	3.00	(-0.74, 6.74)	0.115
	Level of play: Professional/Semi-Pro (vs Youth)	0.82	(-4.36, 6.00)	0.755
	Years of play	0.04	(-0.16, 0.24)	0.687
	Cumulative heading	-0.17	(-0.54, 0.21)	0.382
	Concussion history	0.12	(0.01, 0.23)	0.033
	Age	0.38	(0.27, 0.49)	<0.001
	Education	-0.13	(-0.53, 0.28)	0.543
OTSPSFC	Level of play: High School (vs Youth)	0.17	(-0.81, 1.15)	0.733
	Level of play: College (vs Youth)	0.42	(-0.67, 1.51)	0.450
	Level of play: Professional/Semi-Pro (vs Youth)	0.42	(-1.19, 2.03)	0.608
	Years of play	-0.04	(-0.10, 0.01)	0.106
	Cumulative heading	0.01	(-0.10, 0.12)	0.821
	Concussion history	-0.01	(-0.05, 0.03)	0.698
	Age	-0.03	(-0.06, -0.00)	0.043
	Education	0.08	(-0.04, 0.20)	0.175
SWM Between Errors	Level of play: High School (vs Youth)	2.59	(-0.89, 6.07)	0.144
	Level of play: College (vs Youth)	3.79	(-0.12, 7.71)	0.057
	Level of play: Professional/Semi-Pro (vs Youth)	3.48	(-2.10, 9.05)	0.221
	Years of play	0.10	(-0.11, 0.30)	0.368
	Cumulative heading	-0.24	(-0.64, 0.15)	0.229
	Concussion history	-0.03	(-0.14, 0.08)	0.621
	Age	0.26	(0.14, 0.37)	<0.001
	Education	-0.24	(-0.67, 0.18)	0.263
SWM Strategy	Level of play: High School (vs Youth)	0.24	(-0.29, 0.77)	0.376
	Level of play: College (vs Youth)	0.43	(-0.16, 1.03)	0.155
	Level of play: Professional/Semi-Pro (vs Youth)	0.24	(-0.61, 1.10)	0.573
	Years of play	0.01	(-0.02, 0.05)	0.391
	Cumulative heading	-0.00	(-0.06, 0.06)	0.978
	Concussion history	-0.00	(-0.02, 0.01)	0.592
	Age	0.04	(0.02, 0.06)	<0.001
	Education	-0.02	(-0.09, 0.04)	0.509

Abbreviations: GDS-15 = Geriatric Depression Scale-15, ECog = Everyday Cognition Scale, PAL FAMS = Paired Associates Learning First Attempt Memory Score, PAL TEA = Paired Associates Learning Total Errors Adjusted, BRIEF-A = Behavior Rating Inventory of Executive Function – Adult, MI = Meta Cognition Index, BRI = Behavioral Regulation Index .
